# Downstream or upstream administration of P2Y12 receptor blockers in non-ST elevated acute coronary syndromes: study protocol for a randomized controlled trial

**DOI:** 10.1186/s13063-020-04859-1

**Published:** 2020-11-24

**Authors:** Giuseppe Tarantini, Marco Mojoli, Ferdinando Varbella, Roberto Caporale, Stefano Rigattieri, Giuseppe Andò, Plinio Cirillo, Simona Pierini, Andrea Santarelli, Paolo Sganzerla, Nicoletta De Cesare, Ugo Limbruno, Alessandro Lupi, Roberto Ricci, Carlo Cernetti, Luca Favero, Francesco Saia, Loris Roncon, Valeria Gasparetto, Marco Ferlini, Federico Ronco, Luca Ferri, Daniela Trabattoni, Alessandra Russo, Vincenzo Guiducci, Carlo Penzo, Fabio Tarantino, Ciro Mauro, Alfredo Marchese, Battistina Castiglioni, Alessio La Manna, Matteo Martinato, Dario Gregori, Dominick J. Angiolillo, Giuseppe Musumeci

**Affiliations:** 1 Department of Cardiac, Thoracic, and Vascular Sciences and Public Health, Policlinico Universitario, University of Padova, Via Giustiniani 2, 35128 Padova, Italy; 2 Ospedale Santa Maria degli Angeli, Pordenone, Italy; 3 Ospedali Riuniti, Rivoli, Italy; 4 Ospedale Civile dell’Annunziata, Cosenza, Italy; 5 Sandro Pertini Hospital, Rome, Italy; 6 Azienda Ospedaliera Universitaria Policlinico “Gaetano Martino”, Messina, Italy; 7 University of Naples Federico II, Naples, Italy; 8 Ospedale Bassini, Cinisello Balsamo, Italy; 9 Ospedale Infermi, Rimini, Italy; 10 Hospital of Treviglio Caravaggio, Treviglio, Italy; 11 Policlinico San Marco, Zingonia, Italy; 12 Azienda Ospedaliera Grosseto, Grosseto, Italy; 13 Ospedale Universitario “Maggiore della Carità”, Novara, Italy; 14 Ospedale S. Spirito, Rome, Italy; 15 Ospedale di Treviso ULSS 2, Treviso, Italy; 16 University Hospital of Bologna Sant’Orsola-Malpighi, Bologna, Italy; 17 Hospital Santa Maria della Misericordia, Rovigo, Italy; 18 Casa di Cura Pederzoli, Peschiera, Italy; 19 IRCCS Policlinico San Matteo, Pavia, Italy; 20 Ospedale dell’Angelo, Mestre, Italy; 21 A.O. Ospedale di Lecco, Lecco, Italy; 22 Centro Cardiologico Monzino, Milan, Italy; 23 Sant’Antonio Abate Hospital, Gallarate, Italy; 24 AO-IRCCS Santa Maria Nuova, Reggio Emilia, Italy; 25 Azienda Ospedaliero Universitaria di Ferrara Arcispedale Sant’Anna, Ferrara, Italy; 26 Morgagni-Pierantoni Hospital, Forlì, Italy; 27 Antonio Cardarelli Hospital, Naples, Italy; 28 Anthea Hospital, Bari, Italy; 29 ASST Sette Laghi, Varese, Italy; 30 University Hospital Vittorio Emanuele, Catania, Italy; 31 Division of Cardiology, University of Florida College of Medicine, Jacksonville, FL USA; 32 Division of Cardiology, Azienda Sanitaria Ospedaliera Ordine Mauriziano, Torino, Italy

**Keywords:** Randomized clinical trial, Oral P2Y_12_ inhibitors, Non-ST elevation acute coronary syndrome, Ischemia, Bleeding

## Abstract

**Background:**

The optimal timing to administer a P2Y12 inhibitor in patients presenting with a non-ST elevation acute coronary syndrome remains a topic of debate. Pretreatment with ticagrelor before coronary anatomy is known as a widely adopted strategy. However, there is poor evidence on how this compares with administration of a P2Y12 inhibitor after defining coronary anatomy (i.e., downstream administration). Moreover, there are limited head-to-head comparisons of the two P2Y12 inhibitors—ticagrelor and prasugrel—currently recommended by the guidelines.

**Study design:**

DUBIUS is a phase 4, multicenter, parallel-group, double randomized study conducted in NSTE-ACS patients designed to compare a pretreatment strategy (including only ticagrelor) versus a downstream strategy (including prasugrel or ticagrelor) and to compare downstream prasugrel with downstream ticagrelor. A total of 2520 patients will be randomly assigned to pretreatment with ticagrelor or to no pretreatment. The PCI group of the downstream arm will be further randomized to receive prasugrel or ticagrelor. The two primary hypotheses are that the downstream strategy is superior to the upstream strategy and that downstream ticagrelor is non-inferior to downstream prasugrel, both measured by the incidence of a composite efficacy and safety endpoint of death from vascular causes, non-fatal MI, or non-fatal stroke, and Bleeding Academic Research Consortium (BARC) type 3, 4, and 5 bleedings.

**Conclusions:**

The DUBIUS study will provide important evidence related to the benefits and risks of pretreatment with ticagrelor compared with a strategy of no pretreatment. Moreover, the clinical impact of using downstream ticagrelor compared with downstream prasugrel will be assessed.

**Trial registration:**

ClinicalTrials.gov NCT02618837. Registered on 1 December 2015.

**Supplementary information:**

**Supplementary information** accompanies this paper at 10.1186/s13063-020-04859-1.

## Introduction

Dual antiplatelet therapy (DAPT) including aspirin and a potent P2Y12 receptor inhibitor (prasugrel or ticagrelor) [1, 2] is the current standard of care in patients with non-ST-segment elevation acute coronary syndromes (NSTE-ACS) [3, 4]. In the absence of contraindication, aspirin is routinely administered to all patients as soon as the initial diagnosis is established, irrespective of subsequent management strategy [3]. Conversely, the risk/benefit ratio of the administration of potent P2Y12 receptor inhibitors before coronary anatomy is known (i.e., pretreatment) remains a matter of debate [5–7]. In the ACCOAST trial (A Comparison of Prasugrel at PCI or Time of Diagnosis of Non-ST Elevation Myocardial Infarction) [8], 4033 NSTEMI patients were randomly assigned to receive prasugrel as pretreatment (2 to 48 h before coronary angiography) or to receive prasugrel after angiography, if PCI was planned. Pretreatment with prasugrel did not reduce the 30-day rate of major ischemic events and produced an increase of major or life-threatening bleeding events. Consistently with the observed harm associated to this strategy, current guidelines do not recommend pretreatment with prasugrel in NSTE-ACS (class III, level of evidence B) [1]. To date, there are no head-to-head comparisons between upstream and downstream administration of ticagrelor in NSTE-ACS patients [3]. However, in the PLATO study (A Comparison of Ticagrelor [AZD6140] and Clopidogrel in Patients With Acute Coronary Syndrome) [2], pretreatment with the assigned P2Y12 inhibitor was mandatory per-protocol, and ticagrelor was associated with an early benefit over clopidogrel in invasively managed NSTE-ACS patients irrespective of the timing of angiography [9]. Based on such observations, the European Society of Cardiology (ESC) guidelines have provided divergent recommendations over the years, although most recent guidelines recommend that with class IIa, level of evidence C, pretreatment with ticagrelor be considered as soon as the diagnosis is established [3]. On the contrary, the American College of Cardiology/American Heart Association (ACC/AHA) guidelines are essentially silent on the optimal timing of P2Y12 inhibitor administration and refer to the how the drugs were tested in their pivotal trials [10]. As far as choice of P2Y12 receptor inhibitors in NSTE-ACS patients, both prasugrel and ticagrelor are preferred over clopidogrel when no contraindications are present [3]. Nevertheless, there are no head-to-head comparisons of ticagrelor versus prasugrel specifically in the setting of NSTE-ACS. Recently, 4018 patients with acute coronary syndromes were enrolled in the ISAR-REACT-5 trial (Intracoronary Stenting and Antithrombotic Regimen: Rapid Early Action for Coronary Treatment) and randomized to receive ticagrelor or prasugrel [11]. This trial showed a lower incidence of death, myocardial infarction, or stroke among those who received prasugrel than among those who received ticagrelor, with similar incidence of major bleedings between arms. However, this trial enrolled a mixed population comprising both patients with ST elevated myocardial Infarction and NSTE-ACS. To note, in the subgroup of patients with NSTE-ACS, ticagrelor was to be administered always before angiography, while pretreatment with prasugrel was not allowed. Accordingly, the ISAR-REACT-5 was designed as a comparison of a ticagrelor-based strategy versus a prasugrel-based strategy rather than merely a head-to-head comparison of agents [11]. Currently, guidelines do not provide any specific recommendation in favor of ticagrelor (either upstream or downstream) over prasugrel [1].

Based on such gaps in the evidence, the DUBIUS (Downstream versus Upstream strategy for the administration of P2Y12 receptor Blockers In non-ST elevated acUte coronary Syndromes with initial invasive indication) study was conceived. The primary objectives of this clinical study are (1) to evaluate the impact of a pretreatment strategy based on ticagrelor administration compared to a no pretreatment strategy based on the downstream administration of ticagrelor or prasugrel in a population of NSTE-ACS patients with an indication to undergo invasive evaluation (coronary angiography planned within 72 h from admission) and (2) to evaluate the impact of downstream ticagrelor compared to downstream prasugrel in patients treated by PCI. Study groups will be compared by means of a combined measure of efficacy and safety endpoints (net clinical benefit). In particular, net adverse cardiac events (NACE) will be assessed at 30 days and 12 months. In this paper, we report the study protocol of the DUBIUS study in accordance with the Standard Protocol Items: Recommendations for Clinical Interventional Trials (SPIRIT) guidelines (Additional file 1).

## Methods

### Target population

DUBIUS is a phase 4, prospective, double randomized, active control, parallel arm, multicenter adaptive clinical investigation in a population of NSTE-ACS patients with an indication to undergo initial invasive evaluation, to be conducted at approximately 40 hospitals in Italy, including both academic and non-academic centers. Patients must meet all eligibility criteria (Table 1), including the following: (1) an initial diagnosis of NSTE-ACS (unstable angina or non-ST elevated myocardial infarction), with an onset of symptoms during the previous 24 h and positive troponin-I or troponin-T; (2) an invasive strategy is chosen, meaning that the patient is expected to undergo coronary angiography within 72 h from hospital admission; (3) patient is able to start therapy with a new P2Y12 inhibitor (prasugrel or ticagrelor) or is on a maintenance dose of clopidogrel or ticlopidine and is able to switch to a new P2Y12 inhibitor (prasugrel or ticagrelor); (4) patient is ≥ 18 and < 85 years old. Diagnosis of NSTE-ACS refers to unstable angina or non-ST elevated myocardial infarction (NSTEMI) as previously defined [4]. Exclusion criteria include having contraindications for receiving ticagrelor or prasugrel (e.g., history of stroke or transient ischemic attack, high bleeding risk) and having received a loading dose of a thienopyridine (ticlopidine, clopidogrel, or prasugrel) or a maintenance dose of prasugrel or ticlopidine or ticagrelor within 7 days of entry into the study. An updated list of participating study sites is available online (https://gise.it/studi/gise).

**Table 1:** Eligibility criteria

**Inclusion criteria**	• Age ≥ 18 and < 85. • Non-ST elevated acute coronary syndrome (unstable angina, non-ST elevated myocardial infarction), with an onset of symptoms during the previous 24 h and positive troponin-I or troponin-T. • An initial invasive strategy is chosen (the patient is expected to undergo coronary angiography within 72 h from admission). • Subject is able to start therapy with a new P2Y12 inhibitor (prasugrel or ticagrelor) or is on a maintenance dose of clopidogrel or ticlopidine and is able to switch to a new P2Y12 inhibitor (prasugrel or ticagrelor). • Subject is able to verbally confirm understanding of risks and benefits of dual antiplatelet therapy in coronary acute syndromes, and he/she or his/her legally authorized representative provides written informed consent prior to any clinical investigation related procedure, as approved by the appropriate Ethics Committee. • Patient agrees to comply with follow-up evaluations.
**Exclusion criteria**	**General exclusion criteria** • Known hypersensitivity/contraindication to aspirin, clopidogrel, prasugrel, ticagrelor, heparin, or bivalirudin, or sensitivity to contrast media, which cannot be adequately pre-medicated. • Platelet count < 100,000 cells/mm^3^ or > 700,000 cells/mm^3^, or a white blood cell (WBC) count < 3000 cells/mm^3^ within 7 days prior to index procedure. • Shock. • Have severe hepatic impairment defined as Child-Pugh class C. • Pregnant or nursing subjects and those who plan pregnancy in the period up to 3 years following screening (female subjects of child-bearing potential must have a negative pregnancy test done within 28 days prior to enrollment). • Other medical illness (e.g., cancer or congestive heart failure) or known history of substance abuse (alcohol, cocaine, heroin, etc.) as per physician judgment that may cause non-compliance with the protocol or confound the data interpretation or is associated with a limited life expectancy. • Subject is belonging to a vulnerable population (per investigator’s judgment, e.g., subordinate hospital staff or sponsor staff) or subject unable to read or write. • Currently participating in investigational drug or device trial that has not completed the primary endpoint or that clinically interferes with current trial endpoints. Subject must agree not to participate in any other clinical investigation for a period of 3 years following the index procedure, including clinical trials of medication and invasive procedures. Questionnaire-based studies or other studies that are non-invasive and do not require medication are allowed. **Bleeding risk exclusion criteria** • Prior history of hemorrhagic or ischemic stroke, a transient ischemic attack (TIA), or sub-arachnoid hemorrhage. • History of intracranial neoplasm, arteriovenous malformation, or aneurysm. • Have received fibrinolytic therapy within 48 h of entry or randomization into the study. • Have active pathological bleeding or history of bleeding diathesis. • Have clinical findings, in the judgment of the investigator, associated with an increased risk of bleeding. • Have had recent surgery (within 4 weeks of entry into the study) or are scheduled to undergo surgery within the next 2 months. **Prior/concomitant therapy exclusion criteria** • Have received a loading dose of a thienopyridine (ticlopidine, clopidogrel, or prasugrel) or a maintenance dose of prasugrel or ticlopidine or ticagrelor within 7 days of entry into the study. • Are receiving a GPIIb/IIIa inhibitor (eptifibatide, tirofiban, or abciximab). • Are receiving warfarin or other coumarin derivatives. • Are receiving or will receive oral anticoagulation or other oral antiplatelet therapy (except aspirin [ASA]) that cannot be safely discontinued within the next 3 months. • Are receiving daily treatment with nonsteroidal anti-inflammatory drugs (NSAIDs) or cyclooxygenase-2 (COX2) inhibitors that cannot be discontinued or are anticipated to require > 2 weeks of daily treatment with NSAID or COX2 inhibitors during the study. • Concomitant therapy with a strong cytochrome P-4503A inhibitor or inducer.

### Screening for eligibility

In order to achieve an adequate participant enrolment to reach target sample size, the study will include high-volume sites for referral of NSTE-ACS patients. Participating centers are asked to screen each consecutive patient referred for NSTE-ACS for potential enrolment in the DUBIUS trial.

### Consenting and randomization

**Fig. 1:**
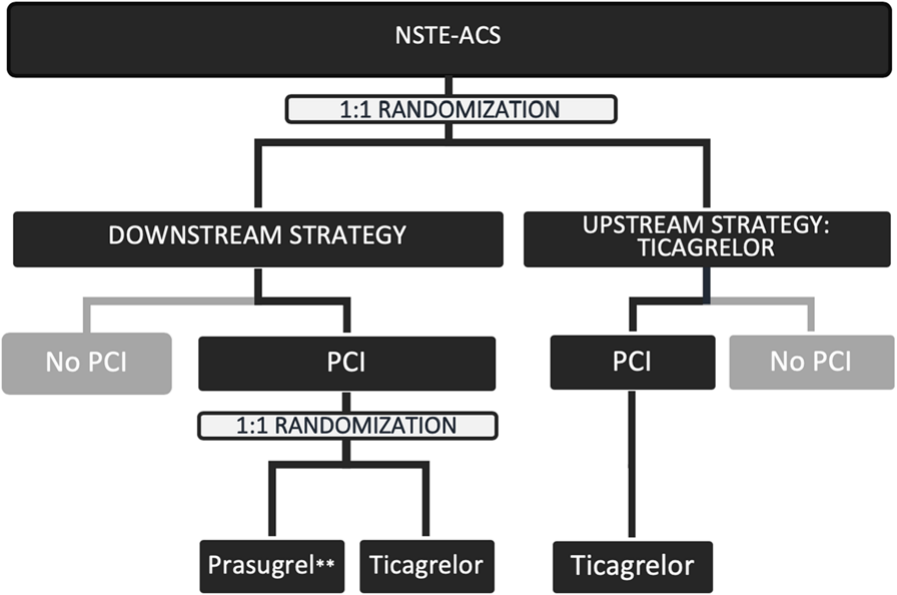
Study design. Patients are randomly assigned (1:1) to downstream P2Y12 receptor blockers (prasugrel or ticagrelor) or to upstream administration strategy (only ticagrelor). The patients of the downstream arm, who will undergo PCI, will be further randomized (1:1) to downstream prasugrel or downstream ticagrelor before the PCI procedure. All randomizations are blocked by age > 75. Prasugrel is to be administered 60 mg oral bolus then 5 mg daily in subjects > 75 years old or with a weight < 60 kg (**)

Investigators of each participating site will be cardiologists or cardiology fellows and will assess eligibility, obtain patient informed consent prior to performing any study procedures, enroll eligible patients, and assign patients to interventions. The informed consent form is reported in Additional file 2. All subjects enrolled will be randomly assigned in a parallel 1:1 fashion to downstream administration strategy of P2Y12 receptor blockers (prasugrel or ticagrelor) or to an upstream administration strategy (only ticagrelor) (Fig. 1). The randomization to the upstream or the downstream arm should occur as soon as possible after admission. The patients of the downstream arm, who will undergo PCI, will be further randomized in a 1:1 fashion to downstream prasugrel versus downstream ticagrelor at the time of PCI. The second randomization (prasugrel versus ticagrelor) will occur after coronary angiography and before the PCI procedure. The allocation sequence of patients into study groups will be computer-generated by a central system located at the coordinating center. Allocation sequence will be concealed to investigators. Investigators will obtain the randomization information of patients enrolled at their site by a dedicated online system (https://redcap.dctv.unipd.it). Enrolled subjects and investigators will not be blinded to study treatments, so unblinding will not occur. In order to avoid imbalances between groups with respect to age, all randomizations will be blocked by age > 75 years or ≤ 75 years. On the consent form, participants will be asked if they agree to use of their data should they choose to withdraw from the trial. Participants will also be asked for permission for the research team to share relevant data with people from the Universities taking part in the research or from regulatory authorities, where relevant. This trial does not involve collecting biological specimens for storage. A centralized online system will be used for randomization. Once randomized, the subjects are considered enrolled in the study and analyzed as the intent-to-treat (ITT) population.

### Study drugs administration

Subjects randomized to the upstream strategy arm will receive a loading dose of ticagrelor (180 mg) at admission as soon as possible after randomization. Those in the upstream arm already receiving a chronic therapy with clopidogrel should switch to ticagrelor therapy, starting with a loading dose of 180 mg of ticagrelor (timing from last clopidogrel dose based on clinician’s judgment). Patients randomized to downstream strategy will not receive any loading dose of P2Y12 receptor blockers before coronary angiography. In both treatment arms, adjunctive aspirin will be administered after randomization (100–300 mg oral or i.v. bolus only in naïve patient; maintenance therapy with ≥ 75 mg daily in all patients) as per standard of care. Patients in the downstream arm already on chronic clopidogrel therapy before randomization will continue maintenance clopidogrel 75 mg daily until coronary angiography, but without receiving any loading dose. The timing of myocardial revascularization (PCI or CABG) in this trial will be based upon judgment of the treating physician. For patients requiring PCI, both ad hoc and deferred procedures are allowed. In the downstream arm, it is recommended to administer the P2Y12 inhibitor as soon as possible after the indication to PCI is formulated and before performing PCI. Moreover, in order to facilitate a complete platelet inhibition before PCI is performed, a deferred PCI (> 2 h from P2Y12 administration) may be preferred. Patients requiring PCI in the downstream arm will receive a loading dose of prasugrel 60 mg or ticagrelor 180 mg according to the second randomization. All subjects treated by PCI will be treated with dual antiplatelet therapy including a minimum of 75 mg of aspirin daily and ticagrelor 90 mg b.i.d. or 10 mg of prasugrel daily (according to randomization) for at least 12 months in line with guideline recommendations. In patients > 75 years of age or with a body weight < 60 kg assigned to receive downstream prasugrel, after a 60-mg loading dose, the daily maintenance dose of prasugrel will be 5 mg. After angiography, if the patient does not have an indication for percutaneous or surgical coronary revascularization, and the diagnosis of NSTE-ACS is confirmed, subjects will maintain (upstream arm) or start (downstream arm) DAPT with ticagrelor based on the benefit of ticagrelor [12], but not prasugrel [13], over clopidogrel in medically treated NSTE-ACS patients. If the initial diagnosis of NSTE-ACS is not confirmed or an alternative diagnosis (e.g., myocarditis, tako-tsubo syndrome) is made, the antiplatelet treatment regimen will be defined at the discretion of the treating physician. In patients who have an indication to undergo coronary artery bypass graft (CABG), antiplatelet therapy will be managed according to current ESC guidelines [3, 14]. In the upstream treatment arm, if ticagrelor is discontinued prior to CABG, resumption of a P2Y12 inhibitor should be considered as soon as considered safe. In the downstream arm, a P2Y12 inhibitor should be started after CABG before patient discharge. Patients who are medically managed as well as those undergoing CABG will be not part of the per-treatment evaluable population but will be part of the intention-to-treat population. Related to allowed or prohibited concomitant medications, the use of GPIIb/IIIa inhibitors prior to randomization is not permitted and is an exclusion criterion. After randomization, a GPIIb/IIIa inhibitor can be used according to local practice. Anticoagulant therapy should be started as per local standard of care as soon as diagnosis of NSTE-ACS is made. Anticoagulants may include unfractionated heparin (UFH), fondaparinux, enoxaparin, and—at the time of PCI—bivalirudin (dosing according to latest ESC guidelines) [3].

### Outcome measures

The primary outcome measure (NACE, net adverse cardiac events) is a composite of death from vascular causes (death from cardiovascular causes or cerebrovascular causes and any death without another known cause), non-fatal MI, or non-fatal ischemic stroke, and BARC type 3, 4, and 5 bleeding. The rationale for choosing this composite endpoint was to use a balanced estimate of the effect regarding irreversible organ damage, that is, MI, stroke, plus an estimate of life-threatening and fatal bleedings. Cardiovascular death includes death with a demonstrable CV cause, or any death that is not clearly attributable to a non-CV cause. To be considered a primary endpoint event, an MI must be distinct from the index event. Definitions of spontaneous, PCI-related, and CABG-related MI will be adjudicated based on the third universal definition of myocardial infarction [15]. Stroke is defined as a rapid onset of a new persistent, neurological deficit that lasts for more than 24 h (excluding hemorrhagic stroke). Bleeding includes major, life-threatening, or fatal events according to BARC type 3, 4, and 5 definitions (Table 2). Secondary outcome measures include death from any cause and stent thrombosis. Stent thrombosis is based on Academic Research Consortium definitions and comprises angiographic or pathological confirmation, as well as clinical determination of stent thrombosis [16]. Secondary safety endpoints include BARC type 2, 3, 4, and 5 bleeding; any TIMI major, life-threatening, and minor bleeding; CABG surgery-related TIMI major and minor bleeding; and non-CABG surgery-related TIMI major and minor bleeding [17, 18].

**Table 2:** Type 3–5 bleeding criteria according to the BARC classification

Type 3	
Type 3a	
Overt bleeding plus hemoglobin drop of 3 to < 5 g/dL (provided hemoglobin drop is related to bleed)	
Any transfusion with overt bleeding	
Type 3b	
Overt bleeding plus hemoglobin drop ≥ 5 g/dL (provided hemoglobin drop is related to bleed)	
Cardiac tamponade	
Bleeding requiring surgical intervention for control (excluding dental/nasal/skin/hemorrhoid)	
Bleeding requiring intravenous vasoactive agents	
Type 3c	
Intracranial hemorrhage (does not include microbleeds or hemorrhagic transformation, does include intraspinal)	
Subcategories confirmed by autopsy or imaging or lumbar puncture	
Intraocular bleed compromising vision	
Type 4: CABG-related bleeding	
Perioperative intracranial bleeding within 48 h	
Reoperation after closure of sternotomy for the purpose of controlling bleeding	
Transfusion of ≥ 5 U whole blood or packed red blood cells within a 48-h period	
Chest tube output ≥ 2 L within a 24-h period	
Type 5: fatal bleeding	
Type 5a	
Probable fatal bleeding; no autopsy or imaging confirmation but clinically suspicious	
Type 5b	
Definite fatal bleeding; overt bleeding or autopsy or imaging confirmation	

### Primary and secondary endpoints

The primary hypothesis will be tested comparing study groups in terms of the 30-day incidence of the combined efficacy and safety primary endpoint (NACE). A list of safety and efficacy endpoints is reported in Table 3.

**Table 3:** Secondary endpoints

Single digit and composite of death from vascular causes (death from cardiovascular causes or cerebrovascular causes and any death without another known cause), MI, stroke, TIA, severe recurrent ischemia, recurrent ischemia, or other arterial thrombotic event	
Death from any cause	
Any stent thrombosis according to the ARC criteria	
Target vessel revascularization (TVR)	
Target lesion revascularization (TLR)	
NACE (net adverse cardiac events) occurred in the period between admission and coronary revascularization defined as a composite of death from vascular causes (death from cardiovascular causes or cerebrovascular causes and any death without another known cause), non-fatal MI, or non-fatal stroke, and BARC type 2, 3, 4, and 5 bleeding	
Compliance to mandated antiplatelet therapy	
BARC type 2, 3, 4, and 5 bleeding (single digit and composite)	
All TIMI major, major life-threatening, and minor bleeding	
All CABG surgery-related TIMI major, minor, and composite of TIMI major or minor bleeding	
Non-CABG surgery-related TIMI major, minor, and composite of TIMI major or minor bleeding	

The primary powered hypotheses include (1) superiority of a downstream administration strategy for P2Y12 receptor blockers over the upstream administration and (2) non-inferiority of prasugrel versus ticagrelor in the PCI group of the downstream strategy arm. The study is powered based on the primary endpoint of incidence at 30 days of the combined primary endpoint. For the comparison of upstream and downstream arms, the intent-to-treat (ITT) population will be considered. The study will provide 80% power to establish the superiority of the downstream strategy on the upstream strategy relative to the primary composite endpoint. These calculations are based on the following assumptions: (1) 8% of patients in downstream arm and 11% of patients in the upstream arm (odds ratio = 1.4) having a primary endpoint event within 30 days from randomization, based on extrapolations of previous data. In particular, in patients with NSTE-ACS pretreated with ticagrelor, the 30-day incidence of a composite efficacy endpoint (comprising cardiovascular death, MI, stroke, TIA, or other arterial thrombotic event) was 6.95%, while the rate of major bleeding was 4.05% [19]. In patients with NSTE-ACS not pretreated with a P2Y12 inhibitor, the observed incidence of the composite efficacy endpoint at 30 days (cardiovascular death, MI, stroke) was 7.2%, while the incidence of non-CABG-related TIMI major bleeds was 0.6% and the incidence of all TIMI major bleeds (CABG-related or non-CABG-related) was 1.5% [8]; (2) the time-to-first event analysis based on a 2-sided log-rank test used at a 2-sided significance level (α) of .05 to assess superiority; (3) a dropout rate of about 10%.

In order to compensate for discrepancies between expected and observed incidence of the primary endpoint, the sample size has been computed using an adaptive approach in three study stages. After each stage, a sample size reassessment will be performed. After having reached the sample size foreseen for each of the three stages, an evaluation using a 95% repeated confidence interval will be used. If the value 0 will be contained in the interval, a sample size reassessment will be performed based on actually observed incidence rates of NACE. Otherwise, data will be passed to the Executive Steering Committee to decide to continue in randomizing patients for the other two analyses or to stop the study. The critical values and the test characteristics of the design were calculated using the Farrington and Manning method [20].

As a second target, non-inferiority of prasugrel versus ticagrelor in the PCI group of the downstream strategy will be considered. Analyses will be performed at the study completion, so no ad interim evaluations are assumed for this study group. Assuming that at least 80% of the patients will have an indication for PCI in the downstream group, based on the above calculations, a sample of about 1000 should be available for randomization in this group. The following assumptions were considered: (i) two-sided non-inferiority test for rejecting the hypothesis that prasugrel and ticagrelor will differ for more than 4% in terms of NACE, (ii) an overall confidence bounds in non-inferiority testing at 0.95 level (α = 0.025), (iii) an overall sample size of 1000 patients (500 prasugrel + 500 ticagrelor), and (iv) an anticipated incidence rate of 5% in downstream ticagrelor and 8.5% in downstream prasugrel and a true difference in incidence rates of 1% between treatment groups [1, 2, 8]. This approach combines the target clinically relevant maximal difference between treatments of 1% with previous information on a relevant distance in terms of effects between treatments (8.5% versus 5%) [21]. This design corresponds to the Palesch and Tilley “futility design” [22, 23], aimed at screening out-of-target therapeutic approaches, in particular in the cardiovascular field [23]. Power calculations have been made using the R-system [24] and gsDesign libraries [25]. For time-to-event variables, survival curves will be constructed using Kaplan-Meier estimates, and log-rank test results will be displayed for descriptive purposes only. Demographics, procedural, imaging, laboratory, exercise testing, quality of life, and diary related data that are not part of the list of endpoints will be displayed for descriptive purposes only.

### Procedures for accounting for missing, unused, or spurious data

All analyses will be based on available data with missing data excluded. Any unused or spurious data will be noted as appropriate in the final report. Analyses will be compared with a multiple imputation approach to detect potential important influences in the study design due to missing values.

### Deviations from the original statistical plan

Any major changes to the statistical plan described above will be documented in amendment to the clinical investigation plan. Less significant changes to the planned analyses will be documented in the final report.

### Public access policy

The full protocol, the participant-level dataset, and the statistical code may be shared or made public for specific purposes or upon participant’s request with approval of the steering committee. The datasets analyzed during the current study are available from the corresponding author on reasonable request.

### Clinical follow-up and assessment of outcomes

**Fig. 2:**
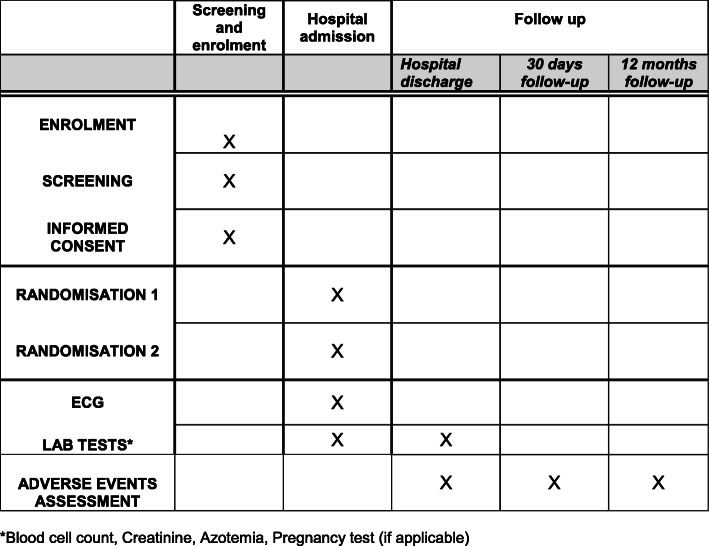
Study flow chart. *Blood cell count, creatinine, azotemia, pregnancy test (if applicable)

The assessment of clinical endpoints and adverse events will take place during hospital stay, at hospital discharge, at 30 ± 7 days (office visit), and at 12 months ± 30 days (office visit) (Fig. 2). Data collection forms are available in the e-CRF at https://redcap.dctv.unipd.it. e-CRF printout is reported in Additional file 3. Investigators at each eligible site will be preliminarily trained by the coordinating center in order to promote data quality related to both clinical data and clinical events. Clinical documentation of each patient experiencing adverse events will be kept at the enrolling sites and made available for the clinical events committee. Investigators at each site will be trained to collect and report any additional reported adverse events and unintended effects of trial interventions or trial conduct by inclusion in the e-CRF or telephone/e-mail contact with the coordinating center, as appropriate. Outcome data of participants who discontinue or deviate from intervention protocols will be collected in a similar manner to those with a satisfactory adherence to study protocol. The responsible person for pharmacovigilance will be available 24/24 h 7/7 days at nucleoricercaclinica@aopd.veneto.it and eudra.vigilance@aopd.veneto.it, telephone +390498218314/4439, mobile +393484720898, and fax +390498217490. Data collection forms will be available in the e-CRF at https://redcap.dctv.unipd.it. The e-CRF printout is available in the Trial Master File and in each center in the appropriate section of Investigator’s Site File.

Investigators are requested to adopt strategies to increase the compliance of patients to study protocol and study treatments. At follow-up visits, investigators will actively monitor the adherence to antiplatelet therapy by means of a dedicated questionnaire included in the e-CRF and, when possible, by direct count of drug tablet consumption. For patients not presenting to follow-up visits, investigators will implement strategies of active promotion of the completeness of follow-up including telephone contact, e-mail/letter contact, or any other suitable procedure, as appropriate.

### Post-trial care

At study completion, each participating subject will receive additional drug treatments or diagnostic investigations according to the treating physician. Subjects who eventually suffered harm from study participation will be cared by the treating physician(s) and may receive compensation upon request to trial sponsor by means of a study-specific insurance policy.

## Study organization

DUBIUS is sponsored by Azienda Ospedaliera di Padova, Padova, Italy. The DUBIUS steering committee is solely responsible for the design and conduct of this study, all study analyses, and the drafting and editing of this and forthcoming manuscripts. Only the steering committee will have full access to the final study dataset, including actual patient data and statistical programming. The steering committee will be responsible for the evaluation of interim analyses and termination of the trial. The steering committee will be responsible for communicating protocol modifications to relevant parties. A clinical endpoints committee will adjudicate primary endpoint events as well as secondary safety events. A statistical analysis center will provide safety data reports and statistical analyses. Safety monitoring will be conducted by a data safety monitoring committee.

Composition, roles, and responsibilities of the steering committee, clinical endpoints committee, and data safety monitoring committee are reported in Appendix 1 (Additional file 4).

### Sponsor contact point

The following is the sponsor contact: Azienda Ospedaliera di Padova, UOC Cardiologia, Via Giustiniani 1, 35128, Padova, Italy. Phone number +390498211844, fax number +390498212309; e-mail giuseppe.tarantini.1@gmail.com

### Monitoring

Coordinating investigator (or designees) will monitor the study over its duration according a pre-specified monitoring plan. The study monitor will visit each site at appropriate intervals to review investigational data for accuracy and completeness and ensure compliance with the protocol. The study monitor may inspect all documents and required records that are maintained by the investigator/site, including medical records (office, clinic, or hospital) for the subjects in this study. The investigator/site will permit access to such records. Source documentation must be available to substantiate proper informed consent procedures, adherence to protocol procedures, adequate reporting and follow-up of adverse events, accuracy of data collected on case report forms, and device information. A monitoring visit sign-in log will be maintained at the site. The investigator and/or research coordinator will be available for monitoring visits. It is expected that the investigator will provide the study monitor with a suitable working environment for review of study-related documents. On-site monitoring for the coordinating center will be performed by the sponsor’s Clinical Research Office while remote monitoring for the remaining centers will be performed by the Department of Cardiac, Thoracic, and Vascular Sciences and Public Health of the University of Padova, by means of its Service for Clinical Trials and Biometrics.

According to ICH E6 (R2) GCP, the frequency of monitoring visits is determined by a risk assessment of the trial performed by the sponsor. The first monitoring visit following initiation of the site and trial commencement will take place within approximately 2 weeks after the inclusion of the first patient. Subsequent monitoring visits will take place every 12 months (for the coordinating center) or every 3 months (for the remaining centers). The interval for monitoring visits may be longer or shorter than stated above, depending on subject enrolment rate, quality issues, trial site compliance, or other trial site issues. Any significant deviation from the planned monitoring timelines will be explained and documented in the monitoring visit report and the monitoring plan amended if appropriate. If the site does not enroll any patients or enrolment has stopped, regular monitoring visits will not be scheduled. If there is an extended gap in trial activity, the monitor should ensure that site staff are appropriately trained when trial activities recommence.

### Auditing

Frequency and procedures for auditing trial conduct are described in the monitoring plan. The audit process is independent from both investigators and the sponsor.

### Regulatory agency inspection

In the event that an investigator is contacted by a regulatory agency in relation to this study, the investigator will notify the coordinating investigator (or designees) immediately. The investigator and research coordinator must be available to respond to reasonable requests and inspection queries made during the inspection process. The investigator must provide the sponsor with copies of all correspondence that may affect the review of the current study. The sponsor will provide any needed assistance in response to regulatory inspections.

## Data handling and record keeping

For the duration of the study, the investigator will maintain complete and accurate documentation including but not limited to medical records, study progress records, laboratory reports, case report forms, signed informed consent forms, device accountability records, and correspondence with the EC and sponsor, adverse event reports, and information regarding subject discontinuation or completion of the study.

### Source documentation

Source documents are defined as original documents, data, and records. Regulations require that the investigator maintains source documents in the subject’s medical records, which confirm the data entered on the case report forms.

### Electronic case report form (e-CRF) completion

Data collection based on source-documented hospital and/or clinic chart reviews will be performed accurately on the e-CRFs by site personnel trained to the protocol and e-CRF completion. Coordinating investigator (or designees) will provide monitoring of e-CRF completion.

### Data entry, coding, security, and storage

Data entry will be performed in a web-based electronic data capture system accessible by username and password provided to each site’s principal investigator or designees. Each subject enrolled in the study will be identified by a numeric code made of study site code and progressive enrolling code; a subject screening and enrolling form will be filed in each study site and stored in a restricted area in a locked cabinet. e-CRF data are stored in a secured server at University of Padua undergoing regular back-up two times per day. In order to promote data quality, range checks for data values have been implemented in e-CRF system; data quality is also routinely performed at fixed timepoint according to monitoring plan both by means of a centralized data quality monitoring system and by manual data quality activities in order to distribute data queries to investigators and collaborators in each study site. Subjects’ privacy will be granted according to EU GDPR. Data management procedures are described in a separate document (e-CRF manual).

### Record retention

The investigator/site will maintain all records pertaining to this study for 3 years following study completion or as otherwise instructed by the coordinating investigator (or designees) or per local regulations if longer.

## Ethical consideration

All subjects must provide written informed consent in accordance with the site’s EC, using an EC-approved informed consent form. Study-specific procedures must not be performed until a signed informed consent has been obtained. The investigator/designee, who have been trained on the protocol, will explain the nature and scope of the study, potential risks and benefits of participation, and answer questions for the subject. If the subject agrees to participate, the informed consent form must be signed and personally dated by the subject or legally authorized representative. The investigator/designee must also sign the informed consent form, prior to subject enrollment. Any additional persons required by the site’s EC to sign the informed consent form must also comply. All subjects are to be fully informed, and study conduct must be in accordance with the World Medical Association Declaration of Helsinki: Ethical Principles for Medical Research Involving Human Subjects.

## Publication policy and authorship eligibility

At the conclusion of the study, a multicenter abstract reporting the primary results will be prepared by the coordinating investigator (in collaboration with the Executive Steering Committee and principal investigators from high enrolling sites) and presented at an annual scientific meeting. A multicenter publication will similarly be prepared for publication in a reputable scientific journal. The publication of the principal results from any single center experience within the study is not allowed until both the preparation and publication of the multicenter results. Following analysis and presentation of the primary endpoint results, active participation of all Executive Steering Committee members and investigators from high enrolling sites will be enthusiastically solicited for data analysis and abstract and manuscript preparation. Submission of all abstracts and publications regarding the primary endpoint and secondary endpoints from the study requires approval. Any person involved in writing or important review of the manuscripts related to the trial will be considered eligible for authorship. It is not expected the use of professional writers for the preparation of manuscripts related to the manuscript.

## Registration

DUBIUS is registered on www.clinicaltrials.gov website with the registration number: NCT 02618837. All items from the World Health Organization Trial Registration Data Set are accessible on https://clinicaltrials.gov/ct2/show/study/NCT02618837. As per Italian and European applicable legislation, DUBIUS is registered on the European Union Drug Regulating Authorities Clinical Trials Database with the EudraCT number: 2015-000993-37.

## Protocol version

The present manuscript reports version 4.0 (June 7, 2016) of study protocol.

## Trial status

DUBIUS is actively recruiting participants. First participant has been enrolled on December 14, 2015. Recruitment is expected to be complete in the fourth quarter of 2020. Current protocol version is 4.0 approved on June 7, 2016.

## Supplementary Information

**Additional file 1.** SPIRIT checklist – DUBIUS study protocol.

**Additional file 2.** Informed consent form.

**Additional file 3.** Case report form

**Additional file 4.** Appendix 1

**Additional file 5.** National Medicines Agency approval

## Data Availability

Not applicable. The manuscript reports a study protocol and does not contain clinical data.
