# Supplementary material for: Downstream or upstream administration of P2Y12 receptor blockers in non-ST elevated acute coronary syndromes: study protocol for a randomized controlled trial
Source: Trials. 2020 Nov 24;21:966. doi: 10.1186/s13063-020-04859-1 (PMC7686679; doi:10.1186/s13063-020-04859-1)
Supplement: Supplementary file 2 — Additional file 2. Informed consent form. [file 13063_2020_4859_MOESM2_ESM.doc]

**FOGLIO INFORMATIVO**

*Azienda Ospedaliera di Padova – Clinica Cardiologica*

**STUDIO “DUBIUS”**

**(Confronto tra strategia downstream e upstream nella somministrazione di bloccanti del recettore P2Y12 in sindrome coronarica acuta senza sopraslivellamente del tratto ST [NSTEACS] con una indicazione invasiva iniziale)**

Gentile Signora / Gentile Signore,

Le proponiamo di partecipare a uno studio promosso dall’Azienda Ospedaliera di Padova – Clinica Cardiologica, che si propone di valutare e confrontare diverse strategie di trattamento con farmaci antitrombotici (cioè antiaggreganti o anticoagulanti) in pazienti affetti da angina instabile o da infarto (senza sopraslivellamento del tratto ST).

Per svolgere questa ricerca, avremmo bisogno della Sua collaborazione.

Prima che Lei decida se partecipare, è importante che abbia tutte le informazioni sul perché questo studio viene fatto e che cosa Le viene chiesto. Può conservare questo foglio informativo e mostrarlo a persone di Sua fiducia (familiari, amici, il Suo medico di medicina generale) che possano aiutarLa a prendere una decisione. Nell’ultima pagina troverà anche i contatti di una persona che Lei può contattare per qualsiasi chiarimento o spiegazione Le dovesse servire.

Nel caso Lei acconsentisse a partecipare, Le verrà chiesto di firmare il Modulo per l’espressione del consenso informato alla partecipazione allo studio e il Modulo di consenso al trattamento dei dati personali.

Le ricordiamo che, anche se accetterà di partecipare, potrà comunque ritirare il Suo consenso in ogni momento, senza dover fornire alcuna motivazione e senza subire alcun tipo di penalizzazione**.**

**1. Che cosa si propone questo studio?**

L'obiettivo di questo studio è quello di confrontare l’efficacia e la sicurezza di diverse strategie di trattamento con farmaci antiaggreganti e anticoagulanti in pazienti affetti da angina instabile o da infarto (senza sopraslivellamento del tratto ST). I farmaci considerati da questo studio rientrano tra quelli attualmente in uso nella corrente pratica clinica.

**2. Quali sono le caratteristiche di questo studio?**

Questo studio è promosso e coordinato dall’Azienda Ospedaliera di Padova – Clinica Cardiologica. Questo studio prevede per tutti i pazienti l’assegnazione casuale (cosiddetta randomizzazione) a uno dei due bracci di studio principali:

- Braccio “upstream” (cioè somministrazione “a monte”): in questo braccio di studio il paziente riceverà al più presto una duplice terapia antiaggregante con aspirina e un inibitore del recettore P2Y12 (ticagrelor), a prescindere dalla eventuale necessità di eseguire di un’angiografia coronarica e un’angioplastica coronarica.
- Braccio “downstream” (cioè somministrazione “a valle”): in questo braccio di studio il paziente riceverà al più presto un trattamento con aspirina, mentre il trattamento con un farmaco inibitore del recettore P2Y12 (prasugrel o ticagrelor) sarà iniziato dopo eventuale angiografia coronarica coronarica (con l’eventuale eccezione dei pazienti con indicazione a bypass aorto-coronarico precoce). Inoltre, i pazienti sottoposti ad angioplastica che appartengono al cosiddetto braccio downstream, verranno assegnati casualmente (randomizzati) a due ulteriori bracci di studio, che prevedono l’assunzione di uno solo tra i seguenti inibitori del recettore P2Y12, il ticagrelor e il prasugrel.

**3. Cosa comporta la mia partecipazione allo studio?**

La partecipazione allo studio comporterà l’assunzione dei farmaci di studio per i successivi 12 mesi. Inoltre, la sua condizione clinica dovrà essere rivalutata dagli investigatori dello studio mediante visita ambulatoriale dopo 30 giorni circa e dopo 1 anno circa dall’ingresso nello studio.

**4. Quali benefici posso aspettarmi?**

I farmaci oggetto di studio (prasugrel e ticagrelor) rientrano tra le terapie comunemente somministrate in caso di angina instabile o da infarto (senza sopraslivellamento del tratto ST). Pertanto, non sono prevedibili per il singolo paziente specifici vantaggi relativi all’inclusione nello studio rispetto ai pazienti con le stesse patologie ma non inclusi nello studio e trattati secondo la corrente pratica clinica.

**5. Quali sono i rischi e/o i disagi derivanti dalla partecipazione a questo studio?**

- I farmaci di studio (prasugrel e ticagrelor) sono potenti antiaggreganti piastrinici. In quanto tali, essi aumentano il rischio di sanguinamenti, anche gravi. Tuttavia, tali farmaci sono comunemente somministrati ai pazienti con angina instabile o infarto poiché hanno complessivamente un comprovato beneficio in tali pazienti, in quanto ne riducono la mortalità e la possibilità di reinfarto rispetto ai pazienti non trattati o trattati con antiaggreganti piastrinici meno potenti.

- Questo studio implica la necessità in donne in età fertile di prevenire possibili gravidanze mediante adeguati metodi anticoncezionali.

**6. Cosa mi succederebbe se decidessi di non partecipare?**

La partecipazione allo studio è del tutto volontaria e se Lei decidesse di non partecipare Le sarebbero comunque garantite le migliori cure possibili.

**7. Quali garanzie esistono a tutela dei partecipanti allo studio?**

Il protocollo di questo studio è stato redatto in conformità alle norme di Buona Pratica Clinica dell’Unione Europea e alla Dichiarazione di Helsinki, ed è stato approvato dal Comitato Etico per la Sperimentazione Clinica (CESC) della Provincia di Padova.

**8. È previsto un rimborso spese per la mia partecipazione?**

La partecipazione allo studio non comporta per Lei alcun costo aggiuntivo diretto.

**9. Sarà tutelata la riservatezza dei dati personali?**

In accordo con le norme di buona pratica clinica e con il Dlgs 196/2003 (e successive modifiche e/o integrazioni) sarà garantita la riservatezza dei Suoi dati personali, così come descritto nel modulo “Informativa e consenso al trattamento dei dati personali”, che Le sarà chiesto di firmare.

**10. Posso essere informato dei risultati della ricerca?**

Se lo desidera, alla fine dello studio potrà essere informato dei risultati ottenuti.

**11. Chi posso contattare per ulteriori informazioni?**

Potrà contattare il dr Giuseppe Tarantini presso la Clinica Cardiologica – Azienda Ospedaliera di Padova. Tel. 0039 049 8211844/2322.

**MODULO PER L’ESPRESSIONE DEL** **CONSENSO INFORMATO**

lo sottoscritto dichiaro di aver ricevuto spiegazioni esaurienti in merito alla richiesta di partecipazione allo studio STUDIO “DUBIUS” (Confronto tra strategia downstream e upstream nella somministrazione di bloccanti del recettore P2Y12 in sindrome coronarica acuta senza sopraslivellamente del tratto ST [NSTEACS] con una indicazione invasiva iniziale) secondo quanto riportato nel foglio informativo qui allegato, copia del quale mi è stata consegnata in data ___________________.

Dichiaro di aver potuto discutere tali spiegazioni, di aver avuto modo di porre tutte le domande che ho ritenuto necessarie e di aver ricevuto in merito risposte soddisfacenti.

Accetto dunque liberamente di partecipare a questo studio, avendo compreso i rischi ed i benefici che esso implica.

Comprendo inoltre che riceverò una copia di questo documento, firmato e datato.

Acconsento / Non acconsento che si comunichi al mio medico di medicina generale quanto a me spiegato sul significato della ricerca cui prenderò parte.

Sono stato inoltre informato del mio diritto ad avere libero accesso alla documentazione relativa alla sperimentazione e alla valutazione espressa dal Comitato Etico.

**PARTECIPANTE**

Nome e cognome: ____________________________________ Data: ______________________

Firma: ________________________________________

**RAPPRESENTANTE LEGALE** *(Eliminare quando non è pertinente)*

Nome e cognome: ____________________________________ Data: ______________________

Firma: ________________________________________

**MEDICO (O RICERCATORE) CHE HA PRESENTATO LO STUDIO**

Io sottoscritto dichiaro di aver spiegato lo studio in modo completo al partecipante e certifico che, al meglio delle mie conoscenze, egli/ella ha compreso la natura e le richieste correlate alla partecipazione a questo studio.

Dichiaro inoltre di aver consegnato al partecipante un originale del modulo di consenso informato, firmato e datato.

Nome e cognome: ____________________________________ Data: ______________________

Firma: ________________________________________
