# Supplementary material for: Downstream or upstream administration of P2Y12 receptor blockers in non-ST elevated acute coronary syndromes: study protocol for a randomized controlled trial
Source: Trials. 2020 Nov 24;21:966. doi: 10.1186/s13063-020-04859-1 (PMC7686679; doi:10.1186/s13063-020-04859-1)
Supplement: Supplementary file 3 — Additional file 3. Case report form [file 13063_2020_4859_MOESM3_ESM.pdf]

Patient Screening

Record ID ________________________________

Screening date ________________________________
(Date informed consent has been signed)

Birth date ________________________________

Inclusion / Exclusion Criteria and First Randomization

Enrolment date (i.e. when informed consent has been signed) ________________________________
(i.e. when informed consent has been signed)

Age ________________________________

Confirm age
◯ >=75 years old
◯ < 75 years old

Inclusion criteria

	Yes	No
Age >= 18 AND < 85	◯	◯
Non ST elevated acute coronary syndrome (unstable angina, non ST elevated myocardial infarction), with an onset of symptoms during the previous 24 hours	◯	◯
An initial invasive strategy is chosen (the patient is expected to undergo coronary angiography within 72 h from admission)	◯	◯
Subject is able to start therapy with a new P2Y12 inhibitor (prasugrel or ticagrelor) OR is on a maintenance dose of clopidogrel or ticlopidine and is able to switch to a new P2Y12 inhibitor (prasugrel or ticagrelor)	◯	◯
Subject is able to verbally confirm understanding of risks and benefits of dual antiplatelet therapy in coronary acute syndromes and he/she or his/her legally authorized representative provides written informed consent prior to any Clinical Investigation related procedure, as approved by the appropriate Ethics Committee	◯	◯
Patient agrees to comply with follow-up evaluations	◯	◯

General Exclusion criteria

	Yes	No
Know hypersensitivity/contraindication to aspirin, clopidogrel, prasugel, tricagrelor, heparin or bivalirulin, or sensitivity to contrast media, which can't be adequately pre-medicated.	○	○
Platelet count< 100,000 cells/mm3 or >700,000 cell/mm3, or a white blood cell (WBC) count < 3,000 cell/mm3 within 7 days prior to index procedure.	○	○
Shock	○	○
Have severe hepatic impairment defined as Child Pugh Class C	○	○
Pregnant or nursing subjects and those who plan pragnancy in the period up to 3 years following screening. (Female subjects of child-bearing potential must have a negative pregnancy test done within 28 days prior to enrollment)	○	○
Other medical illness (e.g., cancer or congestive heart failure) or known history of substance abuse (alcohol, cocaine, heroin etc.) as per physician judgment that may cause non-compliance with the protocol or confound the data interpretation or is associated with a limited life expectancy.	○	○
Subject is belonging to a vulnerable population (per investigator's judgment, e.g., subordinate hospital staff or sponsor staff) or subject unable to read or write.	○	○

Currently participating in investigational drug or device trial that has not completed the primary endpoint or that clinically interferes with current trial endpoints. Subject must agree not to participate in any other clinical investigation for a period of three years following the index procedure, including clinical trials of medication and invasive procedures. Questionnaire-based studies, or other studies that are non-invasive and do not require medication are allowed.	○	○

Bleeding Risk Exclusion Criteria

	Yes	No
Prior history of hemorrhagic or ischemic stroke, a transient ischemic attack (TIA), or sub-arachnoid hemorrhage.	○	○
History of intracranial neoplasm, arterovenous malformation, or aneurysm.	○	○
Have received fibrinolytic therapy within 48 hours of entry or randomization into the study.	○	○
Have active pathological bleeding or history of bleeding diathesis.	○	○
Have clinical findings, in the judgment of the investigator, associated with a high risk of bleeding.	○	○
Have had recent surgery (within 4 weeks of entry into the study) or are scheduled to undergo surgery within the next 2 months.	○	○

Prior/Concomitant Therapy Exclusion Criteria

	Yes	No
Have received a loading dose of a thienopyridine (ticlopidine, clopidogrel or prasugrel) or a maintenance dose of prasugrel or Ticlopidine or Ticagrelor within 7 days of entry into the study.	○	○
Are receiving a GPIIb/IIIa inhibitor (eptifibatide, tirofiban, or abciximab)	○	○
Are receiving warfarin or other coumarin derivatives.	○	○
Are receiving or will receive oral anticoagulation or other oral antiplatelet therapy (except aspirin [ASA]) that cannot be safely discontinued within the next 3 months.	○	○
Are receiving daily treatment with nonsteroidal anti-inflammatory drugs (NSAIDs) or cyclooxygenase-2 (COX2) inhibitors that cannot be discontinued or are anticipated to require >2 weeks of daily treatment with NSAID or COX2 inhibitors during the study.	○	○
Concomitant therapy with a strong cytochrome P-4503A inhibitor or inducer.	○	○

Screening failure

○ True
○ False
(True if eligibility criteria are not satisfied)

Strategy

○ UPSTREAM
○ DOWNSTREAM

Randomization date

____

Demographics, Medical History and Clinical Presentation

Gender ○ M ○ F

Height ______________________
((cm); (round to nearest whole number); (measured anytime during hospitalization))

Weight ______________________
((kg); (round to nearest whole number); (measured anytime during hospitalization))

Clinical presentation and risk stratification

Symptom onset ______________________
(Note: must be within 24 h before enrollment (according to inclusion criteria))

ACS Type ○ Unstable angina ○ Non ST Elevation MI

Heart rate ______________________
(bpm)

Systolic blood pressure ______________________
(mmHg)

Sign of CHF at presentation ○ Yes ○ No

Killip Class
○ 1 (no congestion signs)
○ 2 (mild pulmonary congestion signs)
○ 3 (acute pulmonary edema)
○ 4 (cardiogenic shock)

	Yes	No	Unknown
History of Hypertension	○	○	○
History of Hyperlipidemia	○	○	○
History of Smoke	○	○	○
History of Diabetes Mellitus	○	○	○
Family History of Premature Coronary Artery Disease	○	○	○
Prior coronary stenosis ≥ 50%	○	○	○
ST deviation ECG	○	○	○

If Yes History of Smoking, how often: ○ Current (within last week) ○ Former

If Yes History of Diabetes Mellitus, method of medical treatment
☐ Diet only
☐ Insulin
☐ Oral Tx
(check all that apply)

Type of ST deviation	◯ ST-segment depression<br>◯ T-wave inversion<br>◯ Transient ST-elevation
Prior Myocardial Infarction	◯ Yes<br>◯ No
Prior Percutaneous Coronary Intervention	◯ Yes<br>◯ No
Prior Coronary Artery Bypass Graft Surgery	◯ Yes<br>◯ No
History of Prior Angina	◯ Yes<br>◯ No
History of Congestive Heart Failure (CHF)	◯ Yes<br>◯ No
NYHA Class	◯ 1<br>◯ 2<br>◯ 3<br>◯ 4
Prior Nonhemorrhagic Stroke	◯ Yes<br>◯ No
Prior Hemorrhagic Stroke	◯ Yes<br>◯ No
History of Dyspnea	◯ Yes<br>◯ No
Chronic obstructive pulmonary disease (COPD)	◯ Yes<br>◯ No
History of Chronic Renal Insufficiency	◯ Yes<br>◯ No<br>(GFR < 60 ml/min/1,73/m2 for >=3 months)
Patient is currently on dialysis	◯ Yes<br>◯ No
History of Cardiac Rhythm/Rate Disturbances	◯ Yes<br>◯ No<br>(Ventricular Tachycardia or Fibrillation, Atrial Fibrillation or flutter, Defibrillator implant, Pacemaker implant)
Specify	☐ Ventricular Tachycardia or Fibrillation<br>☐ Atrial Fibrillation or flutter<br>☐ Defibrillator implant<br>☐ Pacemaker implant
≥ 2 Anginal events in prior 24h	◯ Yes<br>◯ No
Aspirin in prior 7 days	◯ Yes<br>◯ No
Elevated cardiac biomarkers	◯ Yes<br>◯ No

First Troponin I / T
◯ Positive
◯ Negative

Baseline hematocrit
____
(% (ex. "45.3" or "35.41"))

Creatinine level
____
(mg/dL; (ex. "1.2" or "1.73"))

Creatinine clearance
____
(mL/min; Estimated with the Cockcroft-Gault formula)

Prior vascular disease
◯ Yes
◯ No

Cardiac arrest ad admission
◯ Yes
◯ No

TIMI Risk Score ________________________________

Grace Risk Score ________________________________

Crusade Risk Score ________________________________

Medications

PRE-ADMISSION MEDICATIONS (ongoing medications before first medical contact [FMC])

	Yes	No
Aspirin	◯	◯
Clopidogrel	◯	◯
Ticlopidine	◯	◯
Beta Blocker	◯	◯
Calcium Channel Blocker	◯	◯
ACE Inhibitor	◯	◯
Angiotensin Receptor Blocker	◯	◯
Insulin	◯	◯
Oral diabetes medications	◯	◯
Statin	◯	◯
PPI	◯	◯

IN-HOSPITAL MEDICATIONS (medications between first medical contact [FMC] and hospital discharge)

Aspirin
◯ Yes
◯ No

Aspirin loading dose performed
◯ Yes
◯ No

Specify Aspirin loading dose (mg)
____
(mg)

Aspirin loading dose date/time
____

Aspirin maintenance dose (>=1 mantainance dose during hospital stay)
◯ Yes
◯ No

Clopidogrel
◯ Yes
◯ No

Clopidogrel loading dose performed
◯ Yes
◯ No

!!!!! COMPLETE PROTOCOL DEVIATION FORM !!!!

Clopidogrel loading dose date/time
____

Reason of Clopidogrel loading dose
____

Specify Clopidogrel loading dose
◯ 300 mg
◯ 600 mg

Clopidogrel maintenance dose (>=1 maintenance dose during hospital stay)
◯ Yes
◯ No

Clopidogrel discontinuation during hospital stay
◯ Yes
◯ No

Reason for clopidogrel discontinuation during hospital stay
◯ Switch to Ticagrelor/Prasugrel
◯ Other reason

Ticlopidine
◯ Yes
◯ No

Ticlopidine loading dose performed
◯ Yes
◯ No

Specify Ticlopidine loading dose (mg)
____
(mg)

Ticlopidine loading dose date/time
____

Ticlopidine maintenance dose (>=1 maintenance dose during hospital stay)
◯ Yes
◯ No

Ticlopidine discontinuation during hospital stay
◯ Yes
◯ No

Reason for Ticlopidine discontinuation
◯ Switch to Ticagrelor / Prasugrel
◯ Other reason

Beta Blocker
◯ Yes
◯ No

Calcium Channel Blocker
◯ Yes
◯ No

ACE Inhibitor
◯ Yes
◯ No

Angiotensin Receptor Blocker
◯ Yes
◯ No

Insulin
◯ Yes
◯ No

Diabetic Oral Medication
◯ Yes
◯ No

Statin
◯ Yes
◯ No

PPI
◯ Yes
◯ No

Heparin (note: excluding periprocedural heparin)
◯ Yes
◯ No

Heparin loading dose
◯ Yes
◯ No

Specify Heparin loading dose
____
(U)

Heparin loading dose date/time
____

Heparin continuous infusion
◯ Yes
◯ No

GP-IIbIIIa inhibitors (note: excluding periprocedural GP-IIbIIIa inhibitors)

◯ Yes
◯ No

GP-IIbIIIa inhibitors loading dose performed

◯ Yes
◯ No

GP-IIbIIIa inhibitors loading dose date/time

____

GP-IIbIIIa inhibitors i.v. infusion

◯ Yes
◯ No

Study Drugs

Ticagrelor

Ticagrelor	◯ Yes<br>◯ No
Ticagrelor loading dose performed	◯ Yes<br>◯ No
Specify Ticagrelor loading dose	________________________
Ticagrelor loading dose date/time	________________________
Ticagrelor maintenance dose (>= 1 maintenance dose during hospital stay)	◯ Yes<br>◯ No
Ticagrelor premature discontinuation occurred (in-hospital)?	◯ Yes<br>◯ No
Specify reason for ticagrelor premature discontinuation (in-hospital)	________________________

Prasugrel

Prasugrel	◯ Yes<br>◯ No
Prasugrel loading dose performed	◯ Yes<br>◯ No
Prasugrel loading dose date/time	________________________
Prasugrel maintenance dose (>=1 maintenance dose during hospital stay)	◯ Yes<br>◯ No
Specify Prasugrel loading dose	◯ 60 mg<br>◯ 30 mg
Specify reason for 30 mg loading dose of Prasugrel	________________________
!!!!! complete protocol deviation form !!!!!	
Specify Prasugrel maintainance dose	◯ 10 mg<br>◯ 5 mg
Specify reason for 5 mg maintenance dose of Prasugrel	
Prasugrel premature discontinuation occurred (in-hospital)?	◯ Yes<br>◯ No
Specify reason for prasugrel premature discontinuation (in-hospital)	________________________

Coronary Angiography

Coronary angiography performed?	◯ Yes<br>◯ No
Reason for not performing coronary angiography	◯ After enrollment, patient has switched from invasive strategy to conservative strategy<br>◯ Death (before performing angiography)<br>◯ Other (specify)
Specify other reason for not performing coronary angiography	______________________________
Date/Hour coronary angiography start	______________________________

CAD extension	◯ 1 Vessel Disease<br>◯ 2 Vessel Disease<br>◯ 3 Vessel Disease<br>◯ Left main involvement (requiring intervention), irrespective of CAD extension<br>◯ Subclinical atherosclerosis (absence of functionally or angiographically significant lesions)<br>◯ Intact coronary arteries (no lesions angiographically)
Vascular approach	☐ radial<br>☐ femoral<br>☐ other
Vascular access closure device	◯ Yes<br>◯ No
Vascular access closure device result	◯ Success<br>◯ Unsuccess
Therapeutic indication after coronary angiography	◯ PCI<br>◯ CABG<br>◯ Hybrid procedure (PCI + CABG)<br>◯ Medical therapy only
PCI scheduled	◯ During current hospital admission<br>◯ During subsequent hospital admission
CABG scheduled	◯ During current hospital admission<br>◯ During subsequent hospital admission

Procedure: PCI

PCI performed
◯ Yes
◯ No

Date/Hour PCI start ____________________

PCI target vessel(s)
☐ LM
☐ LAD
☐ LCx
☐ RCA
☐ Venous graft
☐ Arterial graft

Procedure sucess
◯ Yes
◯ No

BMS: n. stent implanted ____________________

DES: n. stent implanted ____________________

	Yes	No
Treatment of bifurcation lesion(s)	◯	◯
Rotablator	◯	◯
Basal TIMI flow 0/1	◯	◯
No-reflow	◯	◯
Procedural complications	◯	◯
Complete revascularization	◯	◯
Final worsening of TIMI flow (from 2-3 to 0-1 at any treated lesion)	◯	◯

Specify complete revascularization
◯ Single procedure
◯ Multiple procedures

Periprocedural GPIIb/IIIa

Periprocedural GPIIb/IIIa inhibitors
◯ Yes
◯ No

Loading dose during PCI
◯ Yes
◯ No

Type of loading dose during PCI
◯ i.v.
◯ intracoronary

GPIIbIIIa inhibitors infusion
◯ Yes
◯ No

GPIIbIIIa inhibitors suspended immediately after PCI
◯ Yes
◯ No

Periprocedural Unfractionated Heparin

Periprocedural Unfractionated Heparin
◯ Yes
◯ No

Units administered during PCI
____

Protamine sulphate administered
◯ Yes
◯ No

Reason
◯ Major bleeding (BARC 3-5)
◯ Facilitate hemostasis
◯ other

Periprocedural LMWH

Periprocedural LMWH
◯ Yes
◯ No

Specify type of periprocedural LMWH and dose
____

Periprocedural Bivalirudin

Periprocedural bivalirudin
◯ Yes
◯ No

Bivalirudin periprocedural loading dose
◯ Yes
◯ No

Specify loading dose (mg)
◯ Yes
◯ No

Bivalirudin periprocedural infusion
◯ Yes
◯ No

Procedure: CABG

CABG performed
- ◯ Yes
- ◯ No

Date/Hour CABG start __________________________________

Anticoagulant therapy between coronary angiography and CABG
- ☐ None
- ☐ Unfractioned heparin
- ☐ Low molecular weight heparin
- ☐ Fondaparinux
- ☐ Bivalirudin
- ☐ Warfarin/NOACs

Antiplatelet agent(s) discontinued before CABG
- ◯ Yes
- ◯ No

Specify
- ☐ Aspirin
- ☐ Clopidogrel
- ☐ Ticlopidine
- ☐ Prasugrel
- ☐ Ticagrelor

Specify date / hour P2Y12 inhibitor discontinuation __________________________________

Bridging with GPIIbIIIa performed
- ◯ Yes
- ◯ No

Antiplatelet therapy re-started after CABG
- ◯ Yes
- ◯ No

Date / hour antiplatelet therapy re-started after CABG __________________________________

Which drugs re-started
- ☐ ASA
- ☐ Clopidogrel
- ☐ Ticlopidina
- ☐ Prasugrel
- ☐ Ticagrelor

Procedure success
- ◯ Yes
- ◯ No

N. grafts implanted (Total) __________________________________

N. arterial grafts __________________________________

	Yes	No
Emergency CABG for failed PCI	◯	◯
Complete revascularization	◯	◯
Procedural complications	◯	◯

Hospital Discharge

Discharge status
- ◯ Alive
- ◯ Dead

Death from vascular causes (death from cardiovascular causes or cerebrovascular causes and any death without another known cause)
- ◯ Yes
- ◯ No

Which vascular causes
- ◯ Stent thrombosis
- ◯ Fatal myocardial infarction
- ◯ Cerebrovascular event
- ◯ Other cardiovascular event

Date of discharge ______________________________

Date of death ______________________________

Discharge modality
- ◯ Home
- ◯ Transfer to another department
- ◯ Transfer to another hospital

Final diagnosis
- ◯ Initial UA/NSTEMI diagnosis confirmed
- ◯ Initial UA/NSTEMI diagnosis excluded

Please specify final diagnosis (if different from UA/NSTEMI) ______________________________
(e.g. myocarditis, Tako-tsubo...)

THERAPY AT THE TIME OF HOSPITAL DISCHARGE

	Yes	No
Aspirin	◯	◯
Clopidogrel	◯	◯
Ticlopidine	◯	◯
Prasugrel	◯	◯
Ticagrelor	◯	◯
Beta blocker	◯	◯
Calcium Channel Blocker	◯	◯
Insulin	◯	◯
oral diabetes medications	◯	◯
Statin	◯	◯
PPI	◯	◯
Warfarin / NOACs	◯	◯
LMWH	◯	◯

Dual anti platelet therapy at discharge?
- ◯ Yes
- ◯ No

Expected time dual antiplatelet therapy administration
- ◯ 1 month
- ◯ < 6 months
- ◯ =< 12 months
- ◯ > 12 months
- ◯ indeterminate

Follow-up

Adverse events from last follow up

Death
◯ Yes
◯ No

Death from vascular causes (death from cardiovascular causes or cerebrovascular causes and any death without another known cause)
◯ Yes
◯ No

Which vascular causes
◯ Stent thrombosis
◯ Fatal myocardial infarction
◯ Cerebrovascular event
◯ Other cardiovascular event

Specify ______________________________

Specify cause of non cardiac death ______________________________

Date of death ______________________________

Non Fatal Myocardial Infarction
◯ Yes
◯ No

Date of Non Fatal Myocardial Infarction ______________________________
(IF MULTIPLE EVENTS: DATE OF FIRST EVENT)

Non fatal stroke
◯ Yes
◯ No

Date of non fatal stroke ______________________________
(IF MULTIPLE EVENTS: DATE OF FIRST EVENT)

TIA (Transitory Ischemic Attack)
◯ Yes
◯ No

Date of TIA ______________________________
(IF MULTIPLE EVENTS: DATE OF FIRST EVENT)

Recurrent myocardial ischemia
◯ Yes
◯ No

Date of recurrent myocardial ischemia ______________________________
(IF MULTIPLE EVENTS: DATE OF FIRST EVENT)

Any stent thrombosis according to the ARC criteria

	Yes	No
Possible Stent thrombosis	◯	◯
Probable Stent thrombosis	◯	◯

Definite Stent thrombosis ◯ ◯

Date possible stent thrombosis __________________________________

Date probable stent thrombosis __________________________________

Date definite stent thrombosis __________________________________

Other arterial thrombotic event
◯ Yes
◯ No

Specify event
____

Target vessel revascularization (TVR)
◯ Yes
◯ No

Date target vessel revascularization __________________________________
(IF MULTIPLE EVENTS: DATE OF FIRST EVENT)

Target lesion revascularization (TLR)
◯ Yes
◯ No

Date of target lesion revascularization __________________________________
(IF MULTIPLE EVENTS: DATE OF FIRST EVENT)

Premature discontinuation of study drug
◯ Yes
◯ No

Date premature discontinuation of study drug __________________________________

Reason for premature discontinuation of study drug?
◯ Because of adverse event
◯ Becase of patient's ungwillingness to continue
◯ Other

Specify __________________________________

Adherence to study drug
◯ Yes
◯ No
(use of more than 80% of the study medication from last follow up as assessed by investigator)

Major Bleeding (BARC 3-5)
◯ Yes
◯ No

If yes, specify type of bleeding
◯ BARC 3
◯ BARC 4
◯ BARC 5

which BARC 3 bleeding
◯ 3a
◯ 3b
◯ 3c

which BARC 5 bleeding
◯ 5a
◯ 5b

Adverse Event

Adverse Event number ________________________

Event Description ________________________

Worsening of pre-study condition
- ◯ Yes
- ◯ No

If YES, provide details ________________________

Onset date and time ________________________

Stop date and time ________________________

Seriousness criteria
- ☐ Not Serious
- ☐ Death
- ☐ Life threatening
- ☐ Requiring hospitalization or prolongation of hospitalization
- ☐ Results in persistent or significant disability/incapacity
- ☐ Congenital anomaly
- ☐ Important Medical Event

(Select all the applicable)

!!!!!!!!! Please complete JRO SAE form !!!!!!!!!!!!!!!

Severity
- ◯ Mild
- ◯ Moderate
- ◯ Severe

Action taken
- ☐ None
- ☐ Study drug dose reduced
- ☐ Study drug dose increased
- ☐ Pharmacological treatment
- ☐ Study drug dose temporary interrupted
- ☐ Study drug dose definitely interrupted
- ☐ Surgery
- ☐ Other

Action detail ________________________

Causal relationship with the study drugs
- ◯ NO. there is no reasonable causal relationship
- ◯ YES. there is a reasonable causal relationship

IF there is a reasonable causal relationship
- ◯ Certain
- ◯ Probable
- ◯ Possible

Outcome
- ◯ Recovered/resolved
- ◯ Recovering/resolving
- ◯ Recovered/resolved with sequelae
- ◯ Not recovered/not resolved
- ◯ Fatal
- ◯ Unknown

Provide details ________________________

Reason for drop-out
- ◯ Yes
- ◯ No

Comments

Protocol Deviation

Describe Protocol Deviation ______________________________

PLEASE INSERT First Randomization Reference Data

Record ID (Assigned to the Subject on the Enrollment) __________________________________

Enrollment date __________________________________

Date of the First Randomization __________________________________

Birth date __________________________________

Age __________________________________

The subject is
- ◯ >= 75 years old
- ◯ < 75 years old

Second Randomization (prasugrel or ticagrelor in PCI patients - DOWNSTREAM ARM ONLY)

PCI has been chosen as the initial revascularization strategy	◯ Yes<br>◯ No<br>(click "No" if CABG has been performed as initial treatment before PCI)
Downstream PCI strategy	◯ Ticagrelor<br>◯ Prasugrel
Date 2nd randomization	______________________________
