# Supplementary material for: Downstream or upstream administration of P2Y12 receptor blockers in non-ST elevated acute coronary syndromes: study protocol for a randomized controlled trial
Source: Trials. 2020 Nov 24;21:966. doi: 10.1186/s13063-020-04859-1 (PMC7686679; doi:10.1186/s13063-020-04859-1)
Supplement: Supplementary file 4 — Additional file 4. Appendix 1 [file 13063_2020_4859_MOESM4_ESM.docx]

**APPENDIX 1**

**Composition, roles, and reporting structure of the data monitoring committee.**

The data monitoring committee is independent from the sponsor and is composed by the Principal Investigator of the study, the study statistician and the Service for Clinical Trials and Biometry of the University of Padua

**Composition, roles, and responsibilities of the coordinating centre**

The coordinating centre is the Interventional Cardiology Unit of the University Hospital of Padova, head of the unit is the Principal Investigator, assuming the role of coordinating investigator for the whole study. It is responsible for trial master file, budget administration and contractual issues with individual centres, assistance with international review, board/independent ethics committee applications.

**Composition, roles, and responsibilities of the steering committee**

Sergio Berti, Rino Sardella, Francesco Bedogni, Ferdinando Varbella, Alfredo Marchese, Fabio Tarantino, Daniela Trabattoni, Nicola Signore, Leonardo De Luca, Luigi Oltrona Visconti, Salvatore Ierna, Paola Colombo, Antonio Montinaro, Zoran Olivari, Carlo Cernetti, Giampaolo Pasquetto, Dominick J. Angiolillo are the members of the steering committee. This committee has the responsibility of agreement of final protocol, reviewing progress of study and if necessary agreeing changes to the protocol and/or investigators brochure to facilitate the smooth running of the study, study planning, providing annual report and SUSARSs to competent authority and ethics committee and advice for lead investigators.

**Composition, roles, and responsibilities of the endpoint adjudication committee**

Serafina Valente, Emanuela Piccaluga, Claudio Cavallini, Battistina Castiglioni, Alessio La Manna are members of the committee with the responsibility of assessing endpoints adjudication for every single patient.

**Composition, roles, and responsibilities of the data management/analysis team**

Prof. Giuseppe Tarantini, MD and Prof. Dario Gregori, MA, PhD will have the responsibility of maintenance of trial IT system and data entry, data verification, randomization data management

**Composition, roles, and responsibilities of the other individuals or groups overseeing the trial**

*Co-Investigator*

Dr. Giuseppe Musumeci, MD

*Data safety monitoring board (DSMB)*

All adverse events will be reported to the DSMB and reviewed on an on-going basis throughout the subject enrollment and follow-up period as specified in the charter to ensure the safety of subjects enrolled in this study. The DSMB may request additional information as needed. Based on safety data, the DSMB may recommend that the Steering Committee modify or discontinue the study. All final decisions, regarding study modifications, however, rest with the Steering Committee.

Prof. Giuseppe Tarantini, MD, Prof. Dario Gregori, MA, PhD and Dr. Matteo Martinato, MSc, PhD (the latter being the Sponsors’ RPPV - Responsible Person for PharmacoVigilance) are components of this committee.

*Sponsor's medical expert for the trial*

Prof. Giuseppe Tarantini, MD

*Writing Committee*

Submission of all abstracts and publications regarding the primary endpoint and secondary endpoints from the study requires approval by the Coordinating Investigator after review by the Executive Steering Committee. Sergio Berti, Francesco Bedogni, Francesco Saia, Alfredo Marchese, Ciro Mauro, Marco Mojoli, Luca Favero, Plinio Cirillo, Alberto Menozzi, Ugo Limbruno, Massimo Napodano, Paolo Calabrò, Sabino Iliceto, Dominick J. Angiolillo.

**Informed consent form (patient information sheet)**

Gentile Signora / Gentile Signore,

Le proponiamo di partecipare a uno studio promosso dall’Azienda Ospedaliera di Padova – Clinica Cardiologica, che si propone di valutare e confrontare diverse strategie di trattamento con farmaci antitrombotici (cioè antiaggreganti o anticoagulanti) in pazienti affetti da angina instabile o da infarto (senza sopraslivellamento del tratto ST).

Per svolgere questa ricerca, avremmo bisogno della Sua collaborazione.

Prima che Lei decida se partecipare, è importante che abbia tutte le informazioni sul perché questo studio viene fatto e che cosa Le viene chiesto. Può conservare questo foglio informativo e mostrarlo a persone di Sua fiducia (familiari, amici, il Suo medico di medicina generale) che possano aiutarLa a prendere una decisione. Nell’ultima pagina troverà anche i contatti di una persona che Lei può contattare per qualsiasi chiarimento o spiegazione Le dovesse servire.

Nel caso Lei acconsentisse a partecipare, Le verrà chiesto di firmare il Modulo per l’espressione del consenso informato alla partecipazione allo studio e il Modulo di consenso al trattamento dei dati personali.

Le ricordiamo che, anche se accetterà di partecipare, potrà comunque ritirare il Suo consenso in ogni momento, senza dover fornire alcuna motivazione e senza subire alcun tipo di penalizzazione.

1. Che cosa si propone questo studio?

L'obiettivo di questo studio è quello di confrontare l’efficacia e la sicurezza di diverse strategie di trattamento con farmaci antiaggreganti e anticoagulanti in pazienti affetti da angina instabile o da infarto (senza sopraslivellamento del tratto ST). I farmaci considerati da questo studio rientrano tra quelli attualmente in uso nella corrente pratica clinica.

2. Quali sono le caratteristiche di questo studio?

Questo studio è promosso e coordinato dall’Azienda Ospedaliera di Padova – Clinica Cardiologica. Questo studio prevede per tutti i pazienti l’assegnazione casuale (cosiddetta randomizzazione) a uno dei due bracci di studio principali:

- Braccio “upstream” (cioè somministrazione “a monte”): in questo braccio di studio il paziente riceverà al più presto una duplice terapia antiaggregante con aspirina e un inibitore del recettore P2Y12 (ticagrelor), a prescindere dalla eventuale necessità di eseguire di un’angiografia coronarica e un’angioplastica coronarica.

- Braccio “downstream” (cioè somministrazione “a valle”): in questo braccio di studio il paziente riceverà al più presto un trattamento con aspirina, mentre il trattamento con un farmaco inibitore del recettore P2Y12 (prasugrel o ticagrelor) sarà iniziato dopo eventuale angiografia coronarica coronarica (con l’eventuale eccezione dei pazienti con indicazione a bypass aorto-coronarico precoce). Inoltre, i pazienti sottoposti ad angioplastica che appartengono al cosiddetto braccio downstream, verranno assegnati casualmente (randomizzati) a due ulteriori bracci di studio, che prevedono l’assunzione di uno solo tra i seguenti inibitori del recettore P2Y12, il ticagrelor e il prasugrel.

3. Cosa comporta la mia partecipazione allo studio?

La partecipazione allo studio comporterà l’assunzione dei farmaci di studio per i successivi 12 mesi. Inoltre, la sua condizione clinica dovrà essere rivalutata dagli investigatori dello studio mediante visita ambulatoriale dopo 30 giorni circa e dopo 1 anno circa dall’ingresso nello studio.

4. Quali benefici posso aspettarmi?

I farmaci oggetto di studio (prasugrel e ticagrelor) rientrano tra le terapie comunemente somministrate in caso di angina instabile o da infarto (senza sopraslivellamento del tratto ST). Pertanto, non sono prevedibili per il singolo paziente specifici vantaggi relativi all’inclusione nello studio rispetto ai pazienti con le stesse patologie ma non inclusi nello studio e trattati secondo la corrente pratica clinica.

5. Quali sono i rischi e/o i disagi derivanti dalla partecipazione a questo studio?

- I farmaci di studio (prasugrel e ticagrelor) sono potenti antiaggreganti piastrinici. In quanto tali, essi aumentano il rischio di sanguinamenti, anche gravi. Tuttavia, tali farmaci sono comunemente somministrati ai pazienti con angina instabile o infarto poiché hanno complessivamente un comprovato beneficio in tali pazienti, in quanto ne riducono la mortalità e la possibilità di reinfarto rispetto ai pazienti non trattati o trattati con antiaggreganti piastrinici meno potenti.

- Questo studio implica la necessità in donne in età fertile di prevenire possibili gravidanze mediante adeguati metodi anticoncezionali.

6. Cosa mi succederebbe se decidessi di non partecipare?

La partecipazione allo studio è del tutto volontaria e se Lei decidesse di non partecipare Le sarebbero comunque garantite le migliori cure possibili.

7. Quali garanzie esistono a tutela dei partecipanti allo studio?

Il protocollo di questo studio è stato redatto in conformità alle norme di Buona Pratica Clinica dell’Unione Europea e alla Dichiarazione di Helsinki, ed è stato approvato dal Comitato Etico per la Sperimentazione Clinica (CESC) della Provincia di Padova.

8. È previsto un rimborso spese per la mia partecipazione?

La partecipazione allo studio non comporta per Lei alcun costo aggiuntivo diretto.

9. Sarà tutelata la riservatezza dei dati personali?

In accordo con le norme di buona pratica clinica e con il Dlgs 196/2003 (e successive modifiche e/o integrazioni) sarà garantita la riservatezza dei Suoi dati personali, così come descritto nel modulo “Informativa e consenso al trattamento dei dati personali”, che Le sarà chiesto di firmare.

10. Posso essere informato dei risultati della ricerca?

Se lo desidera, alla fine dello studio potrà essere informato dei risultati ottenuti.

11. Chi posso contattare per ulteriori informazioni?

Potrà contattare il dr Giuseppe Tarantini presso la Clinica Cardiologica – Azienda Ospedaliera di Padova. Tel. 0039 049 8211844/2322.

**GP letter**

Caro Collega,

Il Suo assistito ha acconsentito a partecipare, fornendo il suo consenso informato, a uno studio clinico dal titolo “Studio DUBIUS: Confronto tra strategia downstream e upstream nella somministrazione di bloccanti del recettore P2Y12 in sindrome coronarica acuta senza sopraslivellamente del tratto ST (NSTEACS) con una indicazione invasiva iniziale”

Si tratta di uno studio sperimentale, farmacologico, randomizzato, multicentrico italiano, promosso e coordinato dall’Azienda Ospedaliera di Padova – Clinica Cardiologica.

Questo studio arruolerà circa 2520 pazienti con sindrome coronarica acuta senza sopraslivellamento del tratto ST, con iniziale indicazione invasiva (cioè indicazione iniziale a coronarografia ed eventuale angioplastica coronarica). L'obiettivo di questo studio clinico è quello di confrontare in questa popolazione di pazienti l'impatto di diverse strategie antitrombotiche in uso nella corrente pratica clinica, basate sulla somministrazione dei nuovi antagonisti del recettore P2Y12 (prasugrel e ticagrelor). Lo studio esaminerà inoltre gli effetti della somministrazione della bivalirudina rispetto alla terapia standard con eparina non frazionata nei pazienti NSTEACS che si sottopongono ad angioplastica coronarica e che ricevono potenti agenti antipiastrinici.

Tutti i soggetti arruolati saranno randomizzati in rapporto 1:1 ad una strategia di somministrazione downstream (cioè dopo eventuale angioplastica coronarica) dei bloccanti del recettore P2Y12 (prasugrel o ticagrelor) oppure ad una strategia di somministrazione upstream (cioè subito dopo la diagnosi di NSTEACS; solo ticagrelor). A loro volta i pazienti del braccio della strategia downstream che saranno sottoposti a PCI, saranno randomizzati, in rapporto 1:1, agli agenti antiaggreganti prasugrel oppure ticagrelor. Per tutti i pazienti sottoposti a PCI, sia l'uso di eparina non frazionata (più Anti GPIIbIIIa basato su valutazione clinica) e di bivalirudina sarà permesso al momento della PCI; la scelta dell’anticoagulante al momento della PCI sarà basata sul giudizio clinico (open-label).

Quale stima del beneficio clinico netto nel singolo paziente, verrà considerato un endpoint combinato di efficacia e sicurezza a breve (30 giorni) e medio termine (12 mesi).

Il Suo assistito sarà contattato dagli sperimentatori per l’esecuzione di un follow-up ambulatoriale dopo 30 giorni e dopo 12 mesi dall’arruolamento.

I dati personali del Suo assistito raccolti ai fini dello svolgimento della ricerca saranno trattati nel pieno rispetto delle disposizioni di legge e regolamenti vigenti (D.lgs 196/2003); i risultati dello studio a cui partecipa il Suo assistito potranno essere oggetto di pubblicazione, ma la sua identità rimarrà sempre segreta,

Nome e recapito telefonico del Principal Investigator:

Prof. Giuseppe Tarantini, tel. + 39 049 8211844/2322

RingraziandoLa anticipatamente per la Sua collaborazione e restando a disposizione per qualunque necessità o chiarimento, cogliamo l’occasione per porgerle i nostri migliori saluti.

Prof. Giuseppe Tarantini

Principal Investigator studio DUBIUS
