# Supplementary material for: Downstream or upstream administration of P2Y12 receptor blockers in non-ST elevated acute coronary syndromes: study protocol for a randomized controlled trial
Source: Trials. 2020 Nov 24;21:966. doi: 10.1186/s13063-020-04859-1 (PMC7686679; doi:10.1186/s13063-020-04859-1)
Supplement: Supplementary file 5 — Additional file 5. National Medicines Agency approval [file 13063_2020_4859_MOESM5_ESM.pdf]

Agenzia Italiana del Farmaco
AIFA
Ufficio Ricerca e Sperimentazione Clinica

Roma, 02/09/2015

Azienda Ospedaliera di Padova
Giuseppe Tarantini
Via Giustiniani, 1
35128 Padova
giuseppe.tarantini.1@gmail.com

OBJECT: **EudraCT number clinical study: 2015-000993-37**
Comparison of downstream and upstream strategy in the administration of P2Y12 receptor blockers in acute coronary syndrome without elevated ST [NSTEACS] with an initial invasive indication.

On the basis of the evaluation of the documentation received by this Agency in relation to the trial in question, and under Legislative Decree n. 211 of 24 June 2003, art. 9, paragraph 4, it is communicated that AIFA does not object to the initiation of the study in question, which may therefore be considered authorized.

It should be noted that the clinical trial should be conducted in accordance with the standards of Good Clinical Practice (Ministerial Decree 27 April 1992) and subsequent modifications (Ministerial Decree 15 July 1997 and Legislative Decree 211/2003).

Please note that, in order to fulfil the legal obligations relating to the reporting of SUSARs, from 01 February 2014, the Promoters, or the delegated Contract Research Organizations (CRO), are required to send SUSARs exclusively to Eudravigilance Clinical Trial Module (EVCTM).

"DSUR" annual safety reports should be sent to the address **DSUR_ITA@aifa.mailcert.it**.

The manager
(Sandra Petraglia)
